# Antioxidant Activity and Hemocompatibility Study of Quercetin Loaded Plga Nanoparticles 

**DOI:** 10.22037/ijpr.2020.1101000

**Published:** 2020

**Authors:** Serap Derman, Deniz Uzunoglu, Tayfun Acar, Tulin Arasoglu, Samet Ucak, V Cengiz Ozalp, Banu Mansuroglu

**Affiliations:** a *Bioengineering Department, Chemical and Metallurgical Engineering Faculty, Yildiz Technical University, Esenler, Istanbul, Turkey. *; b *Molecular Biology and Genetics Department, Science and Letters Faculty, Yıldız Technical University, Esenler, Istanbul, Turkey.*; c *Medical Biology Department, Medicine Faculty, Altinbas University, Bakirkoy, Istanbul, Turkey. *; d *Bioengineering Department, Engineering and Architecture Faculty, Konya Food and Agriculture University, Meram, Konya, Turkey.*

**Keywords:** Quercetin, Polymeric nanoparticle, Hemocompatibility, Antioxidant activity, Hemolytic activity

## Abstract

Quercetin (QU) is an important flavonoid compound presenting lots of biological activities, but its application has been limited due to its low aqueous solubility and instability. In this study, conducted to improve these properties of the quercetin, quercetin-encapsulated PLGA nanoparticles were prepared, characterized, and evaluated for antioxidant and hemolytic activity. Nanoparticles were produced by single emulsion solvent evaporation method. Four different process parameters initial QU amount, PVA concentration, PVA volume, and initial PLGA amount were investigated to obtain the nanoparticles which have minimum particle size and maximum entrapment efficiency. Synthesized nanoparticles were evaluated for particle size, entrapment efficiency, and reaction yield. Additionally, antioxidant properties and *in-vitro* hemolytic activity of quercetin loaded nanoparticles with different particle size were also evaluated for the first time in the literature. The antioxidant activity results showed that nanoparticles have different antioxidant activity, depending on the amount of quercetin release from nanoparticles at different particle sizes. The hemolytic activity results show that all nanoparticles exhibited favorable compatibility to red blood cells and no significant hemolytic effect was observed.

## Introduction

**Figure 1:**
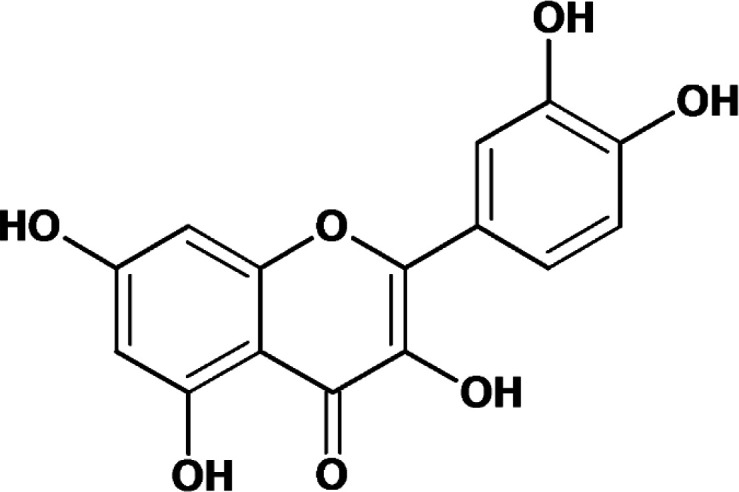
Chemical structure of quercetin (3,3’,4’,5,7-pentahydroxylflavone) (10)

Flavonoids are well known phytochemicals, having anti-oxidative, anti-inflammatory, antimicrobial, and anticancer activities. There are many applications in the field of medicine and pharmacy (1). Quercetin (QU) (Figure 1) is a member of the flavonoid family that is commonly found in vegetables and plants or similar products like apples, and onions. It can be used against many diseases such as ischemic heart disease, atherosclerosis, liver fibrosis, and renal injury. Also pharmacological effects, including antiviral, antitumor, and antioxidant activities of Quercetin have been extensively investigated (2-6). QU, the most abundant flavonoid in the human diet, is known as potent natural antioxidant and scavenger of reactive oxygen and nitrogen species (ROS and RNS) under *in-vitro *and *in-vivo* conditions. It shows potent scavenger activity against superoxide and hydroxyl radicals, nitric oxide, and also peroxynitrite (7-9). However, the use of this flavonoid is limited because of its poor water solubility, low bioavailability, poor permeability, and unstable structure (10-12). We can overcome these problems with entrapment of QU to nanostructure. Thus, its drug solubility, pharmacokinetic, and pharmacological properties can be improved (12). 

Polymeric nanoparticles are colloidal particles smaller than 1 micron (10-1000 nm) diameter and they can be prepared from natural or synthetic polymers. Particle charge, size, shape, and hydrophilicity remain among the most important properties for effective delivery of nanoparticles to the desired target (13, 14). Natural polymers (i.e., proteins or polysaccharides) have not been widely used for this purpose since they vary in purity, and often require crosslinking that could denature the embedded drug (15). Therefore, synthetic polymers have received considerably more attention in this field. The most commonly used synthetic polymers for nanoparticulate system are poly-ε-caprolactone (PCL), poly(lactic acid) (PLA), poly(glycolic acid) (PGA), and their co-polymers, poly(lactide-co-glycolide) (PLGA) (2, 16). As in the comparison with conventional drug delivery systems, nanoparticulate carriers have a lot of advantages (17, 18). Some advantages of nanoparticles are: increased bioavailability, dose proportionality, reduced drug toxicity, smaller dosage form (i.e., smaller tablet), and stable dosage forms of drugs which are either unstable or have unacceptably low bioavailability in non-nanoparticulate dosage forms etc (19-21).

The unique characteristics of nano-sized materials and also its content in case of drug delivery system development studies might result in potentially toxic effects, which make it necessary to evaluate hemocompatibility. Blood compatibility analysis has been established as simple and reliable evaluation method for nanoparticles *in-vitro* (22). Hemolysis can be defined as the disruption of the human red blood cells (erythrocytes) and it is a common parameter to assess biocompatibility of nanoparticles in blood. In this method, erythrocytes damage and cell membrane of erythrocytes disintegrates and hemoglobin molecule leaves from the cell (23, 24).

The aim of this study was to develop quercetin-loaded PLGA NPs of the acceptable size in nano scale with high-quercetin entrapment. For this purpose, quercetin-loaded nanoparticles were prepared with four different processing parameters including initial QU amount, PVA concentration in aqueous phase, PVA volume, and initial PLGA amount. In the single emulsion-solvent evaporation method, these process parameters were screened systematically to improve entrapment efficiency and reaction yield when reducing the particle size. Additionally, antioxidant properties and *in-vitro* hemolytic activity of quercetin loaded nanoparticles with different particle size were also evaluated for the first time in the literature. The obtained results can provide useful information for *in-vitro* and *in-vivo* cell experiments.

## Experimental

*Materials*

Poly(lactic-co-glycolic acid) PLGA (lactide:glicolide=50:50; inherent viscosity 0.45-0.60 dL/g, Mw ~ 38-54 kDa), polyvinyl alcohol (PVA), Quercetin (QU), acetone and ethanol were purchased from Sigma Aldrich (St. Louis, USA), dichloromethane (DCM) was obtained from Ridel de Haen. All the chemicals and solvents were analytical grade. Ultra-pure water was obtained from Millipore MilliQ Gradient system.

*Nanoparticle Preparation*

For preparation of QU loaded nanoparticles, quercetin was dissolved in 750 µL ethanol and PLGA was dissolved in 1.5 mL dichloromethane. Quercetin and PLGA solutions were mixed and added into PVA solution. For emulsifying organic phase with the aqueous phase, 2 min. 80% power sonication was applied and after the sonication the obtained emulsion was added into 0.1% PVA solution. The organic solvent was removed stirring at room temperature at 700 rpm for overnight (F2) (16). 

For purification of the nanoparticles, the nanoparticle suspensions were ultracentrifugated at 9000 rpm, at 4 ˚C for 40 min. The supernatants were removed and nanoparticles were washed thoroughly three times with 35 mL distilled water tree times. The supernatants were collected for the determination of the entrapment efficiency. Nanoparticle pellets were dried using lyophilization at -70 °C, 0.01 mbar pressure.

In this study, F2 nanoformulation was manufactured with amount of 20 mg of QU, 100 mg of PLGA and 4 mL of 3% PVA that was taken as a standard process conditions. Additionally, different process parameters which were used for the synthesis of other nanoparticles were given in Table 1.

*Determination of Entrapment Efficiencies and Reaction Yield*

Determination of entrapment efficiencies was carried out in triplicated, via indirect quantification methods using UV–Vis spectroscopy. The absorbance of the nanoparticles’ supernatants were measured at 374 nm and equivalent concentration was determined via using standard calibration curve which was previously constructed. The entrapment efficiencies and reaction yields were calculated from the following two equations (14).

Entrapment Efficienciy %=Amount of Quercetin determined in the nanoparticle mgAmount of added Quercetin mg                     (1) 

$\mathrm{Reaction Yield}(\%)=\frac{\mathrm{Amount of determined nanoparticle}(\mathrm{mg})}{\mathrm{Amount of quercetin}+\mathrm{PLGA added}(\mathrm{mg})}$                     (2) 

*Particle Size, Polydispersity Index and Zeta Potential Analysis*

The particle size, polydispersity index (PDI), and zeta potential of quercetin-loaded nanoparticles were determined by dynamic light scattering technique using a Zetasizer NanoZS 90. The measurements were performed as follows: 50 µL of the fresh nanoparticle emulsion was diluted to 1.5 mL with distilled water. The measurements were carried out triplicate, at 25 ± 0.1 °C with using 0.8872 cP of viscosity and 1.330 of refractive index for the solutions (25).

*Scanning Electron Microscopy (SEM)*

The morphology of the quercetin-loaded nanoparticles was examined by SEM (A JSM-7001FA Jeol, Japan). For the measurement, dried nanoparticles placed on a double stick tape over aluminum stubs to get a uniform layer and then the particles were coated with gold under vacuum (26). Observation was carried out at the acceleration voltage of 10 kV and at 30.000 x magnification. 

*Fourier Transform Infrared (FT-IR) Spectroscopy*

FTIR spectra of quercetin-loaded nanoparticles were determined using FT-IR (Shimadzu, Japan). The spectra of quercetin-loaded nanoparticles were 16 scans per sample which have ranging from 4000 to 650 cm^-1^ and a resolution of 4 cm^-1^. Background measurement was taken before the measurement of the samples because of the CO_2_ peak (27).

*In-vitro* *release*

In the release study, F2, F6, and F15 NPs which have different particle size, were weighed to contain the same amount of quercetin. *In-vitro* release measurements of quercetin loaded nanoparticles were carried out at 37 °C in phosphate buffer solution at pH = 7.4 as previously reported (28). After a predetermined period (1, 2, 3 4, 24, 48, 72 h), nanoparticle suspensions were ultracentrifugated at 10000 rpm for 15 min at 4 °C. Release medium was fully removed and fresh medium (PBS) was replaced each time. The absorbance values of the supernatants were determined via UV-Vis spectrophotometer at 374 nm. Equivalent concentration was determined using supernatant absorbance and standard calibration curve. 

*Antioxidant Activity*

Antioxidant activity assay with free QU and PLGA NPs were carried out using a previous methodology described by McCord JM and Fridovich I, with minor modifications (29). Free QU and F2, F6 and F15 NPs were solved in 0.1 M phosphate buffer saline (pH 7.4). In a bucket containing a volume of 0.5 mL of each sample was added to 2.45 mL of assay reactive (0.3 mM Xantine, 0,6 mM EDTA, 150 µg/L Nitroblue Tetrazolium (NBT), 400 mM sodium carbonate, 1 g/L BSA, pH 10.2) solution and 50 μL XO enzyme (167 U/L). This system was incubated at 25 °C for 20 min and stopped by adding 1 mL of cupper chloride and immediately absorbance was obtained at 560 nm. Antioxidant activity assay was repeated three times for all samples and expressed as percentage of XO inhibition and calculated as: 

$\mathrm{\% Inhibition}=\frac{\mathrm{Blank absorbance}-\mathrm{Test absorbance}}{\mathrm{Blank absorbance}}\mathrm{x100}$                      (3) 

*Hemolysis of Human Red Blood Cells*

The hemolysis analyses were performed according to Drabkin´s methods by measuring cyanmethemoglobin content of the samples (30). Briefly, whole blood was centrifuged for 1 min at 14000 rpm to collect the blood cells in the pellet. The supernatant was removed and washed with PBS (10 mM, pH 7.4 and 0.85% NaCl) three times. Consequently, 150 µL empty PLGA nanoparticles, quercetin active substances, and quercetin loaded PLGA nanoparticles with different dispersions (F2; entrapment: 95.1 %, 262.6 nm, F6; entrapment: 93.8 %, 869.7 nm, F15; entrapment: 95.5 %, 473.2 nm) were added onto blood cell pellets and incubated for 2 to 6 h at room temperature. Then, the cells were collected by centrifugation. 50 µL supernatants of the samples were mixed with 950 µL Drabkin’s reagent and incubated for 15 min at room temperature. The amount of hemoglobin released from erythrocytes was determined calorimetrically by measuring the absorbance at 540 nm. All the experiments were repeated at least three times with independent blood samples.

## Results and Discussion

Quercetin loaded nanoparticles were synthesized by single emulsion solvent evaporation methods using different process parameters. The particle size, polydispersity index, entrapment efficiency, and the reaction yield of nanoparticles were examined for detailed characterization. After that, antioxidant activity and hemolytic activity of the selected nanoparticles which have different particle size, were investigated as *in-vitro*. 

*Effect of Initial Quercetin Amount*

It is shown that initial amount of the quercetin has no significant effect on particle size, reaction yield, and entrapment efficiency. 

**Figure 2:**
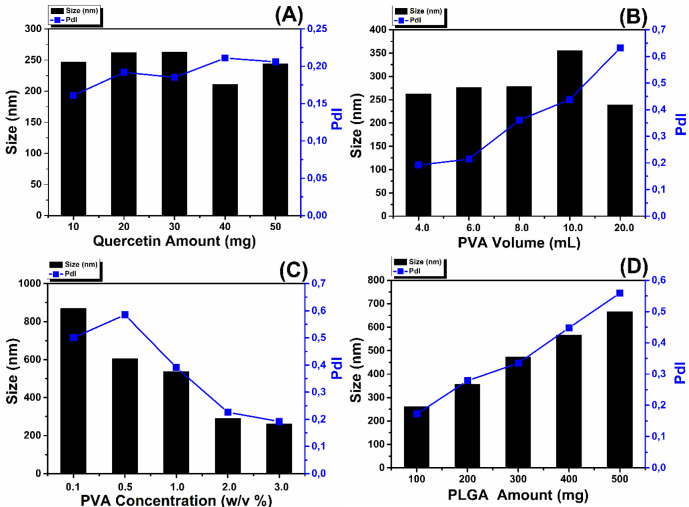
The effect of process parameters on particle size and polydispersity index: effect of Quercetin amount (A); effect of PVA volume (B); effect of PVA concentration (C); effect of PLGA amount (D)

As in the Figure 2A, the mean size of the nanoparticles varied between 211.2 to 262.9 nm by using different amount of the QU. Particle size was firstly stayed constant for 10-30 mg QU, then slightly decreased for 40 and 50 mg QU amount. The polydispersity of nanoparticles synthesized with different QU amount varies from 0.161 to 0.211. This small PDI values (< 0.25) refer to narrow size distribution of the nanoparticles (27).

**Figure 3:**
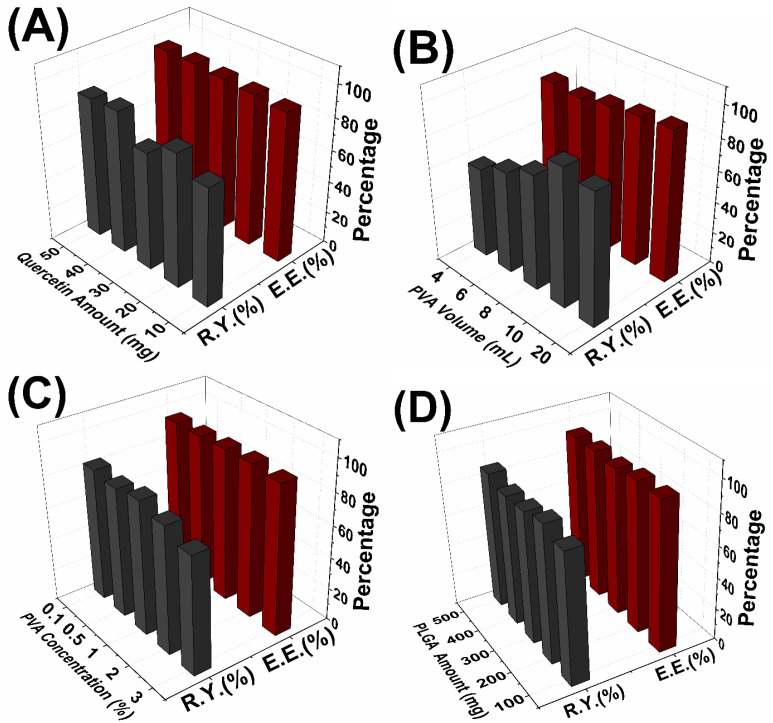
The effect of process parameters on % reaction yield (R.Y.) and % entrapment efficiency (E.E.): (A) effect of Quercetin amount; (B) effect of PVA volume; (C) effect of PVA concentration; (D) effect of PLGA amount

Similarly, increase of the initial Quercetin amount has no significant effect on entrapment efficiency, while increasing the yield of the reaction (Figure 3A). Song *et al*. have shown that increasing amount of antioxidant agent led to decrease of entrapment efficiency with a range of 20% to 40% (31). In our study, this result couldn’t be observed due to the very high entrapment efficiencies which were between 93.4% and 95.1%. Additionally, the reaction yield increased irregularly with increasing of initial QU amount. This situation can be explained in accordance with the literature that, increase of the reaction yield was possibly caused by increase of the QU in the organic phase and it caused more interaction between QU and PLGA molecules (31).

*Effect of PVA Volume*

Figure 2B shows that increasing PVA volume causes increase of the mean diameter of the nanoparticles except for 20 mL PVA volume. Increasing of PVA volume has the same effect with PVA concentration that when PVA volume increases, the amount of PVA increases and it decreases interfacial tension and on the other side it increases viscosity (31, 32). In our study it was shown that in terms of PVA volume, increasing viscosity dominates decreasing interfacial tension thereby causes bigger particle sizes.

It can be understood from the Figure 3B, entrapment efficiencies slightly decreased with the increase of PVA volume. With increasing of total volume, more quercetin dissolves in aqueous phase and this can lead to loss quercetin during the nanoparticle synthesis. This situation occurred because of the amount of the drugs partitioned into the organic phase reduced during the emulsification (33);<e’. 

Additionally, the reaction yields of nanoparticle significantly decreased with increasing of PVA volume. High solubility of QU in the external phase will result in decreased entrapment efficiency and consequently decreased reaction yield. 

*Effect of PVA Concentration*

Figure 2C shows that with the increase of the PVA concentration, the mean diameter decreases and also polydispersity index decreases which indicates narrow size distribution of the nanoparticles. Similar size results have also been reported in the literature (20, 31, 33-35). When PVA concentration increases, interfacial tension decreases which led to important increase in the net shear stress and formation of smaller nanoparticles droplets. On the other hand, increase of PVA concentration causes increase of the viscosity and hence causes bigger size of the droplets. But the decrease of the size of the nanoparticles in this study is a result of domination of decreasing interfacial tension over the increasing viscosity (20, 31). 

With high PVA concentration promoting stability to the emulsion, interfacial tension, the free energy in the interface, and aggregation decrease; thus smaller particles are formed (34). It was found that 3% PVA concentration is the best PVA concentration which was enough to cover the droplets completely that provides smaller particle size. 

PVA concentration has no significant effect on entrapment efficiencies despite change of particle sizes. Additionally, reaction yields are slightly increased with increasing PVA concentration (Figure 3C), and similar result was obtained by Hussein *et al* (36). 

*Effect of Initial PLGA Amount*

In the present study, the obtained results showed that the PLGA concentration is the most important factor affecting both mean particle size and PDI of nanoparticles. Figure 2D shows the increase of the mean diameter of the nanoparticles with the increase of PLGA concentration. Increase of the PLGA amount leads to three different situations. Firstly with the increase of PLGA amount, viscosity of the organic phase increases and this causes decrease of the net shear stress which is a resistance for droplets to be broken by external energy and it also prevents removing PLGA from organic phase into aqueous phase. These situations are concluded with formation of the larger nanoparticles droplets. Secondly, with more PLGA, PVA amount may not be enough to cover the surface of the droplets and this causes the aggregation of the droplets during elimination of the organic solvent (31, 34). Thirdly, with the increase of PLGA amount; greater numbers of polymeric chains are applied to the emulsion and diffusion of the solvent into the aqueous phase becomes hard and this causes aggregation and formation of larger nanoparticles (37). Additionally, PDI values of the nanoparticles varied between 0.172 and 0.559. These results mean that the nanoparticles show narrow size distribution to broadened size distribution with increase of the initial PLGA amount. Figure 3D shows that increasing of the PLGA amount has no significant effect on entrapment efficiencies and reaction yields which are almost same for 100-500 mg PLGA. 

*SEM and FT-IR Analysis*

**Figure 4:**
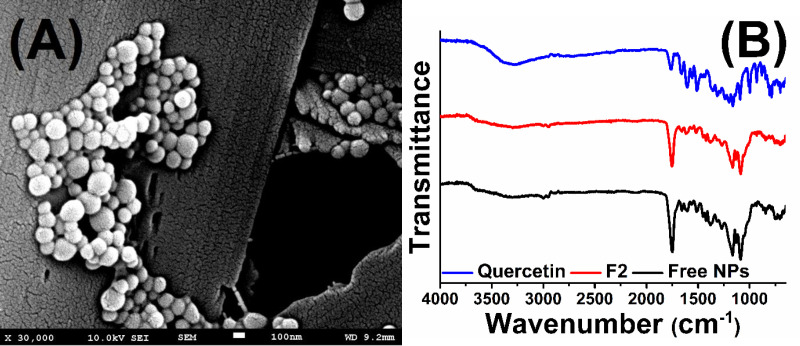
Scanning electron micrographs of quercetin loaded PLGA nanoparticles (A) and FT-IR spectrum of quercetin, free nanoparticles and quercetin loaded nanoparticles (B).

Scanning electron microscopy was used to investigate the morphological properties of quercetin loaded PLGA nanoparticles (F2 formulation). The SEM micrograph was shown in Figure 4A and supported by the DLS results showing that the nanoparticles have a homogeneous distribution with spherical morphology. It is thought that this result is consistent with the literature (38).

ATR FT-IR was used to characterize the surface of the nanoparticles and to determine if the quercetin be adsorbed on the nanoparticle surface. The FT-IR spectra of quercetin, free nanoparticles, and quercetin encapsulated PLGA nanoparticles (results were given for F2) were given in Figure 4B. In the FT-IR spectra, absorption peaks are especially assigned to specific functional groups. The free quercetin showed the main characteristic peak in the region 1655 cm^-1^ due to carbonyl groups (C=O) and the other important peaks are C=C stretching that is observed at 1512 cm^-1 ^and C-O stretching which is observed at 1286 cm^-1^. PLGA polymer gives specific peaks at 1750 cm^-1^ and between 1250-1100 cm^-1^ corresponding to C=O and C-O groups, respectively. When compared to the spectrum of the quercetin loaded nanoparticle with the empty nanoparticle, it was observed that the specific peaks of PLGA did not change and no new peak appeared after the encapsulation of quercetin. The obtained results are in agreement with literature (39). This demonstrated that the quercetin molecules did not adsorb the surface region of the nanoparticles and were successfully encapsulated into the nanoparticles. 

*In-vitro* *Release*

**Figure 5:**
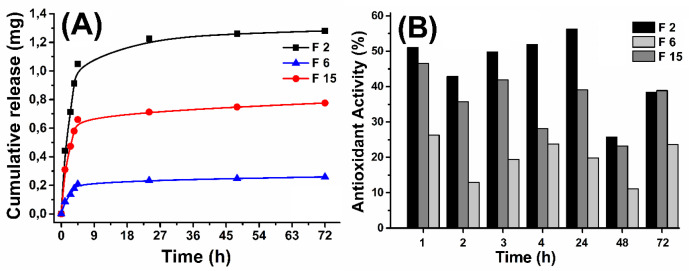
Cumulative release (A) and time dependent antioxidant activity (B) results for F2, F6 and F15 nanoparticles

In order to demonstrate the effect of the particle size on the quercetin release, release studies were performed with different sizes of nanoformulations. For this purpose, F2, F6, and F15 NPs were weighed to include the same amount of quercetin and obtained release pattern was shown in Figure 5A. Nanoparticles at different size between 262.6 and 869.7 nm showed remarkable differences in quercetin release. Since smaller nanoparticles have larger surface area-to-volume ratio and small diffusion path lengths, faster and more quercetin release has been observed for F2 NPs. The obtained results are consistent with literature (40, 41). 

*Antioxidant Activity*

In the formulations F2 (262.6 nm), F6 (869.7 nm), and F15 (473.2 nm) the initial QU amounts were taken equal (20 mg) and the other parameters (PVA volume, PVA and PLGA amount) were changed to be obtained in different sizes nanoparticles. In order to demonstrate the effect of the particle size on the time dependent antioxidant effect, antioxidant activity study was performed for F2, F6, and F15. Time dependent antioxidant activity was investigated by xanthine-xanthine oxidase system. The release mediums of F2, F6, and F15 were used for antioxidant activity assay and the obtained antioxidant activity results were shown in Figure 5B. 

The obtained antioxidant activity results, in agreement with the release study demonstrated the released amount of QU increased with decreasing of the particle size. Consequently, antioxidant activity decreased with increasing of particle size or decreasing amount of released QU. According to literature, antioxidant activity of quercetin has been shown to vary depending on the dosage, it is thought that the antioxidant activity of F2, F6, and F15 NPs influences the dose differences.

*Hemolytic Activity of PLGA*

**Figure 6:**
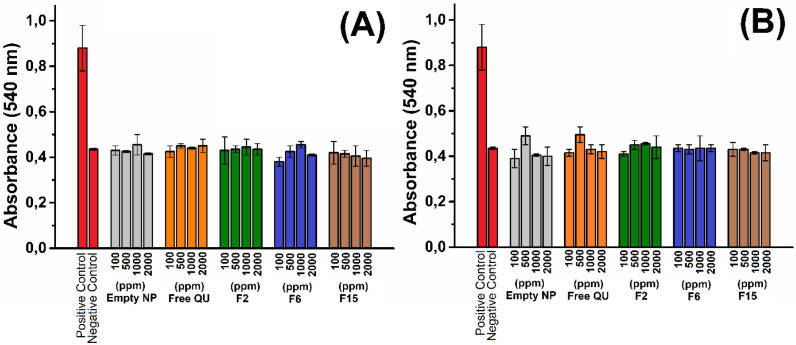
Hemolytic activity in different dispersions and concentration of nanoparticles at 2 h (A) and 6 h (B).

Hemolytic activity of PLGA nanoparticles were evaluated by measuring hemoglobin release from red blood cells incubated with nanoparticles. F2, F6, and F15 NPs at various concentrations 100-2000 ppm were contacted with red blood cells and lysis of the cells was calorimetrically investigated. Hemolytic activities for different NPs and concentrations evaluated up to 6 h incubation. As shown in Figure 6, the absorbance of free QU, empty NPs, F2, F6, and F15 NPs is nearly similar to negative (PBS) control. No hemolysis was observed after nanoparticle administration. Similar results have been obtained in the literature using different drug loaded PLGA nanoparticles (42-44). Surolia *et al* investigate the hemolytic activity of monensin loaded PLGA nanoparticles and it was shown that both empty and monensin loaded PLGA nanoparticles do not have any hemolytic activity (42). In another study Luo *et al* explored the effect of nanoparticle surface functionalization on hemolytic activity. In the study it was shown that when erythrocytes were incubated with negative charged PLGA nanoparticles no hemolysis was observed. Otherwise, when erythrocytes were incubated with positively charged nanoparticles, it was shown that the hemolysis occurred (43). The results obtained our study indicate that the all nanoparticle formulations are compatible with blood and they can be used safely for intravenous administration. 

**Table 1:** Experiments with different process parameters for single emulsion-solvent evaporation method

**Sample**	**Quercetin Amount (mg)**	**PVA** **Volume (mL)**	**PVA Concentration (%)**	**PLGA Amount (mg)**
F1	10	4	3	100
F2	20	4	3	100
F3	30	4	3	100
F4	40	4	3	100
F5	50	4	3	100
F6	20	4	0.1	100
F7	20	4	0.5	100
F8	20	4	1	100
F9	20	4	2	100
F10	20	6	3	100
F11	20	8	3	100
F12	20	10	3	100
F13	20	20	3	100
F14	20	4	3	200
F15	20	4	3	300
F16	20	4	3	400
F17	20	4	3	500

## Conclusion

In the study, PLGA based polymeric nanoparticles were synthesized for design of effective delivery system of antioxidant quercetin molecules which are hydrophobic flavonoid and extremely water insoluble. The prominent features of an entrapment strategy are to enhance the water solubility and sustained antioxidant activity of flavonoids compared to the free form. Nanoparticular controlled release system are administered to humans through oral, nasal, intramuscular or intravenous routes, thus interactions between the nanoparticular system with blood compartments are important for future applications. For this reason, the aim of the present study is to examine the antioxidant activity and hemocompatibility of the nanoparticle systems with different particle size. Different synthesis parameters, which play a critical role in controlling different properties of the nanoparticles, were investigated and their effects on the particle size were demonstrated. 

Three different sized quercetin loaded nanoparticles (F2 →262.6 nm, F6 → 869.7 nm and F15 → 473.2 nm, respectively) and approximately same entrapment efficiency (95.1%, 93.8 %, and 95.5%) were compared with each other in terms of antioxidant activity. Also, we examined free QU, empty NPs, F2, F6, and F15 NPs with different concentrations (100 ppm, 500 ppm, 1000 ppm and 2000 ppm). We observed large differences in both release pattern and antioxidant activity between nanoparticles of different sizes, this is due to the larger surface area/volume ratio of the smaller nanoparticles and hence faster and more quercetin release from them. As in many studies about PLGA nanoparticles (24, 45), we have not found any hemolytic effect of PLGA nanoparticles depending on time. Additionally, its hemocompatible nature of this QU loaded NPs allows a possibility of the safe bio application in wide range. 
